# Transferrin receptor-mediated reactive oxygen species promotes ferroptosis of KGN cells via regulating NADPH oxidase 1/PTEN induced kinase 1/acyl-CoA synthetase long chain family member 4 signaling

**DOI:** 10.1080/21655979.2021.1956403

**Published:** 2021-08-08

**Authors:** Lingzhi Zhang, Fang Wang, Dongmei Li, Yufeng Yan, Hongyan Wang

**Affiliations:** Department of Obstetrics and Gynecology, Cao County People’s Hospital, Shandong, China

**Keywords:** Polycystic ovary syndrome, iron metabolism, ferroptosis, mitophagy

## Abstract

Polycystic ovary syndrome (PCOS) is the most common endocrine disorder in women of reproductive age. Abnormal ovarian folliculogenesis is the main factor responsible for PCOS. Iron metabolism plays a vital role in endocrine disorder. This study aimed to investigate the potentials of iron metabolism in PCOS and the underlying molecular mechanisms. Mice were injected with dehydroepiandrosterone (DHEA) to establish the PCOS model in-vivo. H & E staining was performed for histological analysis; qRT-PCR and western blot were employed to determine the mRNA and protein expressions. Immunofluorescence was used for mitochondrial staining. Cellular functions were detected using CCK-8 and PI staining assays. Ferric ammonium citrate (FAC) activates the transferrin receptor (TFRC), increases the iron content, and suppresses the cell viability of the human granulosa-like tumor cell line (KGN). However, TFRC knockdown suppressed ferroptosis of KGN cells. Iron uptake mediated the activation of NADPH oxidase 1 (NOX1) signaling, which induced the release of reactive oxygen species (ROS) and mitochondrial damage. Moreover, TFRC activated PTEN induced kinase 1 (PINK1) signaling and induced mitophagy; iron-uptake-induced upregulation of acyl-CoA synthetase long chain family member 4 (ACSL4) was required for mitophagy activation and glutathione peroxidase 4 (GPX4) degradation. Additionally, FAC increased iron uptake and suppressed the folliculogenesis in-vivo. In conclusion, TFRC increased the iron content, mediated the release of ROS, activated mitophagy, and induced lipid peroxidation, which further promoted the ferroptosis of KGN cells. Therefore, the inhibitory effects of TFRC/NOX1/PINK1/ACSL4 signaling on folliculogenesis can be a potential target for PCOS.

**Figure:**
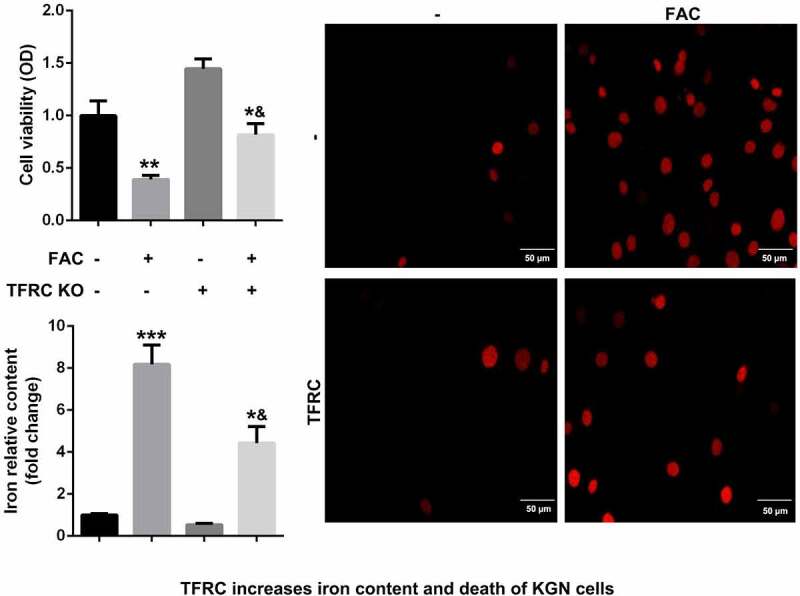

## Introduction

Polycystic ovary syndrome (PCOS) is the most common endocrine disorder in women of reproductive age, with a 9% incidence rate [1]. The clinical symptoms of PCOS are oligomenorrhea, amenorrhea, hirsutism, and infertility [2]. Moreover, PCOS increases the risk of type 2 diabetes mellitus, anovulatory infertility, and insulin resistance [3]. The strategies for treating PCOS mainly focus on treating menstrual irregularities, hirsutism, and androgen resistance, with laparoscopic ovarian drilling being the main technique focused on [4]. However, the long-term outcomes are still unsatisfactory. Dewailly et al. revealed that abnormal ovarian folliculogenesis is the main reason for the initiation and development of PCOS [5]. Hence, understanding the underlying molecular mechanisms is vital.

Ferroptosis is iron- and oxidative stress-dependent regulated cell death, characterized by an increase in iron content, lipid peroxidation, and plasma membrane damage [6]. Iron metabolism, including iron import, export, storage, and turnover, maintains iron homeostasis, which plays a crucial role in regulating sensitivity to ferroptosis [7]. Overexpression of iron responsive element binding protein 2 (IREB2) – a master regulator of iron metabolism – increases cell sensitivity to ferroptosis by inducing iron transportation via activation of the transferrin receptor (TFRC) [8]. Increase of labile iron pool (LIP) induced by heme oxygenase-1 (HMOX1) promotes ferroptosis by catalyzing the degradation of heme to ferrous iron, biliverdin, and carbon monoxide [9]. TFRC, as a key regulator of cellular transferrin-iron uptake, interacts with transferrin to modulate iron import, which further promotes sensitivity to ferroptosis [10]. However, its downregulation suppresses erastin-induced ferroptosis [11]. Wu et al. revealed that TFRC induces cysteine deprivation and regulates system Xc^−^, which transports extracellular cystine to cytoplasm for the exchange of intracellular glutamate [10]. Iron metabolisms collectively participate in endocrine disorders, including diabetes [12], thalassemia [13], cervical cancer [14], and PCOS [15,16]. However, the underlying mechanisms are still unclear.

Ferroptosis suppresses transport of cystine, inducing glutathione depletion and downregulation of phospholipid peroxidase glutathione peroxidase 4 (GPX4). GPX families are classified as selenocysteine-containing GPXs and cysteine-containing GPXs [17]. GPX4 is the only GPX that protects bio membranes from peroxidation damage. However, inactivation of GPX4 via depletion of GSH promotes ferroptosis [18]. There is increasing evidence that GPX4-degradation-induced ferroptosis can be a therapeutic strategy for cancer [19–21]. Moreover, lipophilic radical-traps and iron chelators are the crucial regulators in the genetic depletion of GPX4 and the accumulation of lipid reactive oxygen species (ROS). This study aims to investigate iron metabolism in ferroptosis in PCOS.

## Materials and methods

### Mice

Female C57/BL6 mice were purchased from Nanjing Medical University (Nanjing, China). Mice were maintained in a pathogen-free facility under 12-h light/dark cycles, with free access to water. The mice (8–12 weeks) were randomly divided into three groups: sham group, dehydroepiandrosterone (DHEA) group, and DHEA+FAC group. Mice in the sham group were subcutaneously injected with 1.5 mg/g sesame oil. Mice in the DHEA group were injected with 1.5 mg/kg of DHEA (diluted in 0.1 ml sesame oil). Mice in DHEA+FAC group was injected with 1.5 mg/g of DHEA and 1 mg/kg of FAC. After 21 days, the mice were sacrificed.

This study was approved by the Ethics Committee of Cao County People’s Hospital.

### H & E staining

Ovarian and uterine tissues were collected and fixed in 4% paraformaldehyde and embedded in paraffin. Tissues were sliced and stained with hematoxylin and eosin. The results were visualized with a light microscope (Olympus, Japan).

### Fe-transferrin uptake

Fe-transferrin uptake was detected as described in a previous study [22]. Briefly, rats were intraperitoneally injected with Fe-transferrin. The rats were euthanized; blood was collected by cardiac puncture, and tissues were dissected. Subsequently, Fe-transferrin uptake was detected using a gamma counter.

### Cell culture and transfection

KGN cells were obtained from ATCC, USA. Cells were cultured in DMEM medium supplemented with 10% FBS at 37°C in an atmosphere with 5% CO_2_.

Cells were treated with 100 µM of FAC, 3 µM of RSL3, 100 µM of H_2_O_2_ (36%), or 5 mM of NAC.

Si-TFRC, si-GPX4, si-NOX1, si-PINK1, si-ACSL4, PINK1 knockout (KO), Penta KO, ATG5 KO, p62 OE, Beclin OE, NBR1 OE, and the negative control were purchased from GenePharm, Shanghai. Cells were transfected using Lipofectamine® 2000 (Invitrogen). After 48 transfections, the cells were used for the following experiments.

### qRT-PCR

Total RNAs were collected from tissues and cells using the TRIzol® reagent (Invitrogen). cDNA were synthesized from RNA using the Prime-Script RT Reagent kit (Takara, Japan). PCR was conducted using SYBR green qPCRmaster mix on the ABI PRISM 7500 real-time PCR system. The thermocycling conditions were as followed: 5 min at 95°C, followed by 40 cycles of 95°C for 30 sec and 65°C for 45 sec. GAPDH was used as the loading control. mRNA expression was calculated using the 2^−ΔΔCq^ method. The sequences of the primers used as followed: TFRC forward: 5ʹ-ACCATTGTCATATACCCGGTTCA-3ʹ and reverse: 5ʹ-CAATAGCCCAAGTAGCCAATCAT-3ʹ; Tubulin forward: 5ʹ-AACGAGCTGTGCTACAAGGTC-3ʹ and reverse: 5ʹ-GCGTGGTCGATGAGGAAGA-3ʹ.

### Western blot

Cells were harvested and lysed. Total proteins were collected using the RIPA buffer. The concentration of protein was determined using a BCA kit. The proteins were separated into equal amounts (30 µg) using 12% SDS-PAGE at 120 v. Then, the separated proteins were moved onto PVDF membranes. The membranes were blocked with nonfat milk. The membranes were then incubated with primary antibodies such as anti-TFRC (ab214039, 1:1000, Abcam USA), anti-GPX4 (ab125066, 1:3000, Abcam, USA), anti-NOX1 (ab121009, 1:1000, Abcam, USA), anti-PINK1 (ab216144, 1:1000, Acam, USA), anti-cyto C (ab133504, 1:5000, Abcam USA), anti-Parkin (ab73015, 1:1000, Abcam, USA), anti-Tom20 (ab186735, 1:1000, Abcam, USA), anti-COX II (ab179800, 1:1000, Abcam, USA) and anti-Tubulin (ab6046, 1:500, Abcam, USA) and the next day, with secondary goat-anti-rabbit antibodies (ab6721, 1:2000, Abcam, USA). Subsequently, the bands were visualized using an ECL kit and analyzed using ImageJ v1.52a software.

### Cell viability assay

Cells were plated onto a 96-well plate. The cells were then cultured with the CCK-8 reagent (20 µl/well) and incubated for 4 h at 37°C in air containing 5% CO_2_. The results were determined with a microplate reader at a wavelength of 450 nm.

### Malondialdehyde (MDA), Fe^2+^, reactive oxygen species (ROS), and GSH determination

The levels of MDA, Fe^2+^, ROS, and GSH were determined using specific commercial MDA (Sigma-Aldrich), Iron Assay Kit (Sigma-Aldrich) kits, ROS detection assay kit (Abcam), and GSH assay kit (Beyotime, China) at wavelengths of 532, 593, 490, and 412 nm, respectively.

### Flow cytometry assay

The apoptotic ratio was measured by propidium iodide (PI) double staining (KeyGEN BioTECH, Nanjing, China) as per the manufacturer’s protocol, followed by flow cytometry analysis (BD Pharmingen, San Diego, CA).

The level of BODIPY C11 was determined by flow cytometry using FlowJo 7.6 software.

### Immunofluorescence

Cells were fixed with 4% paraformaldehyde. Then, 0.3% Triton X-100 was added to the cells to control penetrability. The cells were then incubated with primary antibodies, such as anti-PINK1 (ab216144, 1:1000, Acam, USA), anti-Tom20 (ab186735, 1:1000, Abcam, USA), anti-Parkin (ab73015, 1:1000, Abcam, USA) and anti-ASCL4 (PA5-27137, 1:1000, Invitrogen, USA), anti-cyto C (ab133504, 1:5000, Abcam USA), and Hsp60 (ab190828, 1:1000, Abcam, USA) in the shade at room temperature for 1.5 h, followed by incubation with secondary goat-anti-rabbit antibodies (ab6721, 1:2000, Abcam, USA) for 1 h. 4´,6-diamidino-2-phenylindole (DAPI) was used to stain the nuclei.

### Statistical analysis

All data were analyzed using SPSS 22.0 and represented as mean ± SD. Student *t* test was used for analyzing the difference between two groups, and one-way ANOVA was used for multiple group analysis. A result of P < 0.05 was deemed as statistically significant.

## Results

### Enhancement of ferroptosis of KGN cells through activation of iron metabolism by FAC

**Figure 1.**
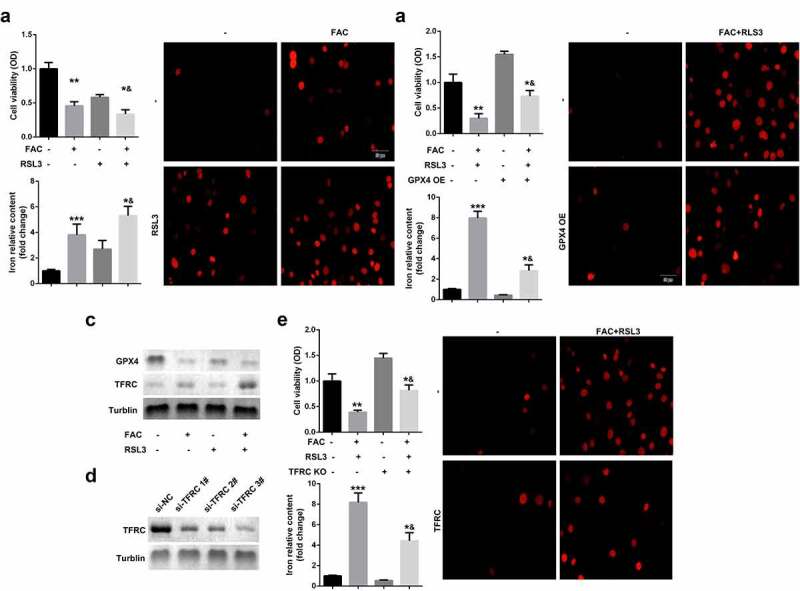
FAC activates iron metabolism to enhance the ferroptosis of KGN cells

Iron metabolism plays an essential role in maintaining cellular functions. Its dysfunction is a key risk factor leading to gynecological diseases including cervical cancer, ovarian cancer, preeclampsia, and PCOS. Previous studies have revealed that FIN56, silica-based nanoparticles, and FAC induce iron metabolism. Among these reagents, the effects of FAC are more significant in inducing iron metabolism and cell death. As shown in Figure 1a, FAC significantly increased iron content, suppressed cell viability of KGN and promoted cell death. FAC combined with RSL3 suppressed the protein expression of GPX4, a key regulator of ferroptosis, and increased TFRC, a transferrin receptor (Figure 1c). To further verify the role of iron metabolism on the ferroptosis of KGN cells, cells were subjected to TFRC knockdown or treatment with GPX4 overexpression plasmids. As shown in Figure 1b, GPX4 expression alleviated the effects of FAC combined with RSL3 and suppressed the release of iron and cell death. The protein expression of TFRC was significantly decreased after transfection with the small interference RNAs, which was more significant in si-TFRC 3# (Figure 1d). Therefore, si-TFRC 3# was used in the experiments whose results are provided below. Moreover, TFRC knockdown significantly suppressed ferroptosis of KGN cells induced by the combination of FAC and RSL3, as well as the release of Fe^2+^ (Figure 1e).

(a) FAC promoted cell death, iron release and ferroptosis of KGN cells. (b) Overexpressed GPX4 inhibited the ferroptosis of KGN cells. (c) FAC increased the protein expression of TFRC and decreased GPX4. (d) Tranfection efficiency of TFRC. (e) Knockdown of TFRC suppressed the ferroptosis of KGN cells. Each experiment was performed in triplicate. **P < 0.01, ***P < 0.001, *^&^P < 0.01.

### Promotion of ferroptosis of KGN cells through iron-uptake-induced activation of ROS signaling

**Figure 2.**
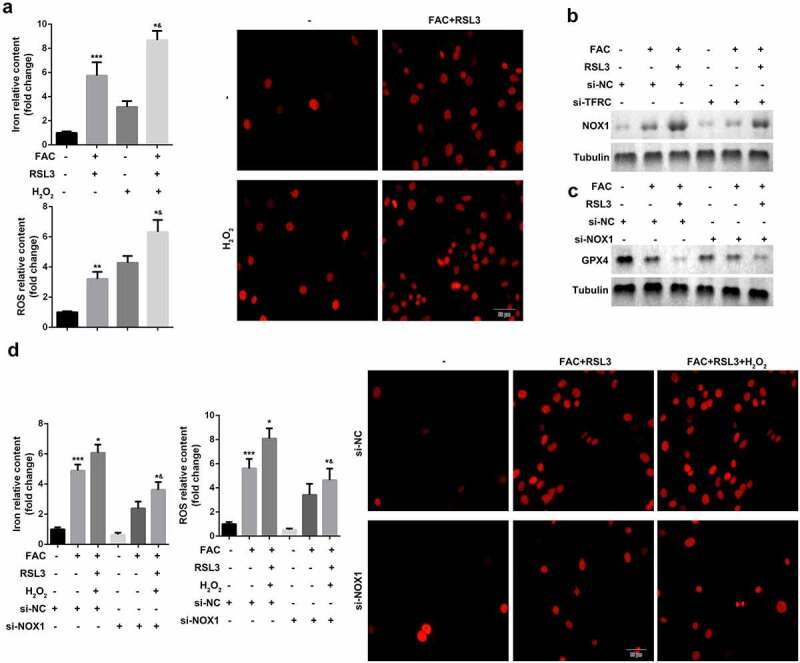
Iron uptake-induced activation of ROS signaling promoted the ferroptosis of KGN cells

Ferroptosis is an iron- and oxidative-stress dependent process, characterized by the accumulation of Fe^2+^ and ROS. We identified the role of ROS pathways in FAC-induced ferroptosis of KGN cells. As shown in Figure 2a, FAC induced cell ferroptosis and upregulation of GPX4 were more obvious in cells treated with a combination of FAC and H_2_O_2_ groups. Moreover, FAC increased the protein expression of NOX1, a major regulator of oxidative stress, which was reversed by the TFRC knockdown (Figure 2b). Additionally, downregulation of NOX1 reversed the effects of FAC, increasing the protein expression of GPX4 (Figure 2c). Furthermore, the downregulated NOX1 decreased the release of Fe^2+^ and reduced the death of KGN cells (Figure 2d).

(a) H_2_O_2_ promoted FAC induced ferroptosis. (b) Iron uptake increased the protein expression of NOX1. (c) Activation of NOX1 decreased the expression of GPX4. (d) NOX1 knockdown suppressed the ferroptosis of KGN cells. Each experiment was performed in triplicate. **P < 0.01, ***P < 0.001, *^&^P < 0.01.

### Enhancement of ferroptosis of KGN through iron-uptake-induced upregulation of PINK1

**Figure 3.**
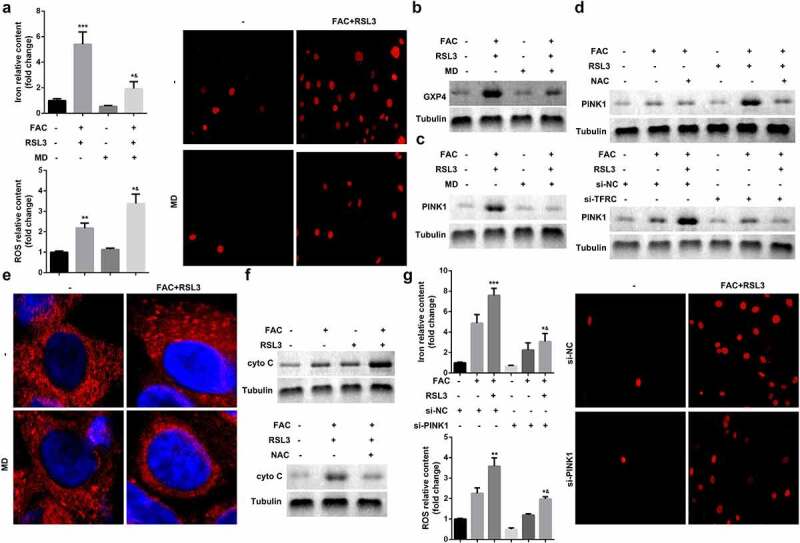
Iron uptake induced upregulation of PINK1 enhance the ferroptosis of KGN

Ferroptosis is characterized by smaller than normal mitochondria with condensed mitochondrial membrane densities, vanishing mitochondrial crista, and ruptured outer mitochondrial membrane. Therefore, we investigated mitochondrial signaling in the ferroptosis of KGN cells. As shown in Figure 3a and b, mitochondria depletion suppressed FAC-induced ferroptosis of KGN cells. Previous studies have revealed that mitochondrial damage increases the release of cytochrome C (cyto C). To further verify the role of mitochondria in ferroptosis, we determined the expression of cyto C. As shown in Figure 3e and f, FAC increased the expression of cyto C, which was alleviated by mitochondria depletion or inhibition by ROS. Additionally, activation of PINK1 signaling, induced by FAC, was diminished by mitochondria depletion (Figure 3c). NAC, (N-acetyl-L-cysteine), which antagonizes the activity of ROS, abrogated the effects of FAC and promoted the downregulation of PINK1 (Figure 3d). Knockdown of PINK1 promotion suppressed the release of ROS and iron content and inhibited cell death (Figure 3g)

(a) Mitochondrial depletion induced the ferroptosis of KGN cells. (b) Mitochondrial depletion suppressed the protein expression of GPX4. (c) Mitochondrial depletion inhibited the expression of PINK1. (d) Knockdown of TFRC or NAC suppressed the expression of PINK1. (e) FRC promoted mitochondrial accumulation. (f) FAC enhanced cyto C release. (g) PINK1 KO suppressed ferroptosis of KGN. Each experiment was performed in triplicate. **P < 0.01, ***P < 0.001, *^&^P < 0.01.

### FAC induced upregulation of PINK1 promoted mitophagy in KGN cells

**Figure 4.**
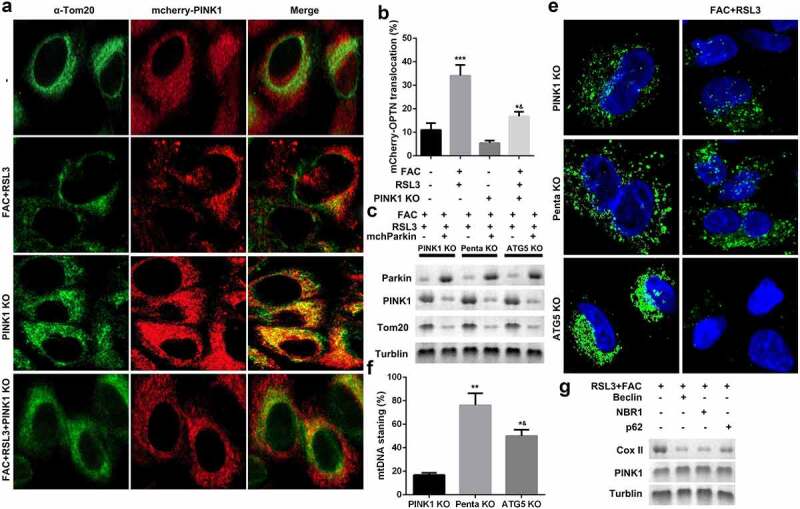
Iron uptake induced upregulation of PINK1 promoted mitophagy in KGN cells

Mitophagy-independent function of PINK1 plays an important role in regulating mitochondrial function and stress response. As shown in Figure 4a and b, FAC recruited mCherry-PINK1 to mitochondria. However, there was nearly no mtDNA in the PINK1 knockdown group; however, penta KO and ATG5 KO still maintained FAC-induced-mitophagy suppressed by mtDNA Parkin (Figure 4c-e). We also determined the expression of cytochrome C oxidase subunit II (CoxII), a mtDNA encoded inner membrane protein. FAC-induced-upregulation of COX II was reduced by penta KO and ATG5 KO (Figure 4f).

(a and b) FAC/RSL3 recruited mCherry-PINK1 to mitochondria. (c-e) Penta KO and ATG5 KO still maintained mtDNA Parkin and suppressed FAC/RSL3 induced mitophagy. (f) FAC/RSL3 increased the protein expression of COX II. Each experiment was performed in triplicate. **P < 0.01, ***P < 0.001, *^&^P < 0.01.

### Importance of ACSL4-dependent lipid metabolism for PINK1-induced mitophagy and ferroptosis of KGN cells

**Figure 5.**
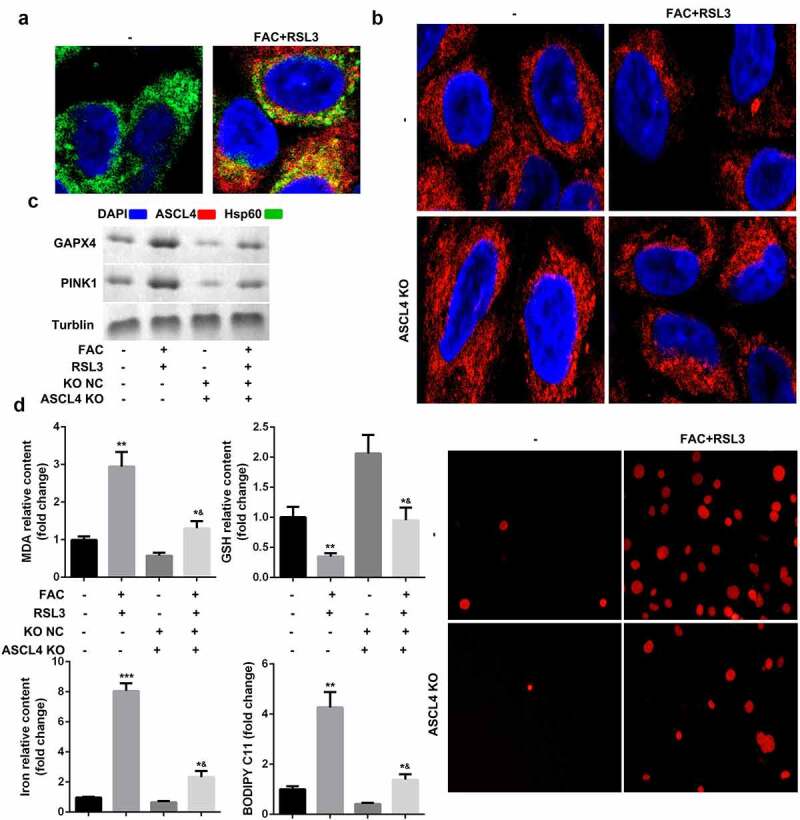
ACSL4-dependent lipid metabolism is required for PINK1-induced mitophagy and ferroptosis of KGN cells

Oxidative-stress-induced lipid peroxidation contributes to mitochondrial dysfunction and cell death. We therefore investigated the potential roles of lipid metabolism in the mitophagy and ferroptosis of KGN cells. ACSL4, a lipid metabolism-associated gene, is reported to promote RSL3-induced ferroptosis. As shown in Figure 5a, FAC promoted the translocation of ACSL4 into mitochondria. ACSL4 knockdown suppressed FAC-induced mitochondrial dysfunction and upregulation of PINK1 and GPX4 (Figure 5b and c). Moreover, downregulated ACSL4 decreased the accumulation of MDA, GSH, iron, and BODIPY C11, and suppressed cell death (Figure 5d).

(a) FAC promoted the translocation of ACSL4 to mitochondria. (b) ACSL4 knockdown suppressed mitochondrial aggregation. (c) ACSL4 knockdown the expression of PINK1 and GPX4. (d) ACSL4 knockdown inhibited the ferroptosis of KGN cells. Each experiment was performed in triplicate. **P < 0.01, ***P < 0.001, *^&^P < 0.01.

### Role of FAC in restoring ovarian function

**Figure 6.**
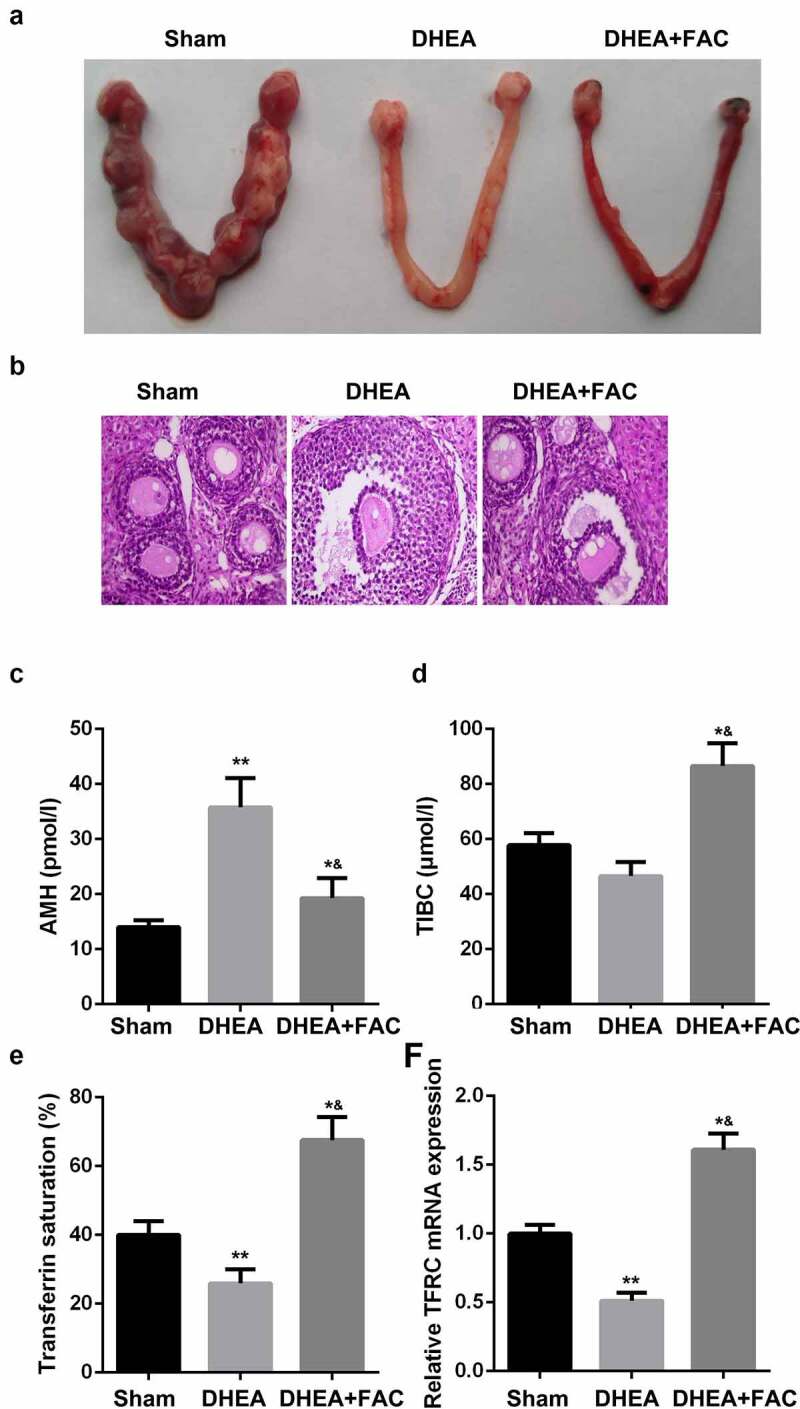
FAC restored the function of ovary

To further verify the nature of iron metabolism when PCOS is present, rats were injected with DHEA to establish an in-vivo model of PCOS. Figure 6a shows the number of embryos implanted after the DHEA injection, which was alleviated by FAC. Moreover, H & E staining results showed that FAC suppressed the formation of polycystic, enlarged ovaries and the thinner granular cell layer induced by the DHEA injection (Figure 6b). Additionally, FAC alleviated the effects of DHEA and modulated the levels of AMH, TIBC, transferrin saturation, and the mRNA expression of TFRC (Figure 6c-f).

(a) FAC increased the number of implanted embryos. (b) FAC restored ovarian function. (c-f) FAC decreased the level of AMH. (d) FAC increased the level of TIBC. (e) FAC promoted transferrin saturation. (e) FAC increased the mRNA expression of TFRC. **P < 0.01. *^&^P < 0.01.

## Discussion

This study reveals that iron metabolism has a role in the initiation and progression of PCOS. Our study revealed that iron-mediated mitophagy promotes the ferroptosis of KGN cells by activating TFRC/PINK1 signaling. FAC induced the activation of TFRC; TFRC also increased the release of iron, contributing to the accumulation of ROS and overexpression of PINK1. The activation of PINK1-signaling induced mitophagy and lipid peroxidation, which induced the downregulation of GPX4 and ferroptosis of KGN cells. Additionally, in vivo assay further demonstrated that FAC suppressed the progression of PCOS. Therefore, targeting TFRC/PINK1/ACSL4 signaling can be a promising therapy for PCOS.

Iron metabolism including iron uptake, storage, utilization, and efflux is intensively involved in ferroptosis [23]. Iron metabolism involves the following:

(1) Iron chelators suppress cell ferroptosis [24] (2) The process of ferroptosis is frequently accompanied by an increase in cellular labile iron [25] (3) The accumulation of Fe^2+^ promotes sensitivity to ferroptosis promoters [26] (4) The augmentation of heme and non-heme iron can directly induce ferroptosis [27] (5) Various iron-containing enzymes, e.g., NOXs, are involved in the process of ferroptosis; and

(6) Iron-induced ROS accumulation by Fenton reaction facilitates lipid peroxidation [28].

The activation of transferrin receptor (TFRC) – an iron uptake protein – induced by the degradation of nitrogen fixation 1 (NFS1) which is a cysteine desulfurase, enhances sensitivity to ferroptosis [29]. In this study, FAC exposure induced the accumulation of iron content, which represented a failure of iron metabolism in KGN cells. TFRC increased after FAC treatment and promoted the sensitivity of KGN cells to ferroptosis via increasing iron-induced oxidative stress and mitophagy. Moreover, TFRC induced oxidative modification and recruited NOX1 to PINK1 to enhance mitochondrial aggregation and mitophagy, and cytochrome c release to cytosol, followed by activation of ACSL4, which is a key regulator of lipid peroxidation as well as a promoter of ferroptosis [30]. Thereupon, the mitochondria function acts as a platform connecting the iron-elevated ROS with ferroptosis via PINK1, promoting sensitivity to intracellular ROS.

Autophagy – especially selective autophagy – plays an essential role in maintaining cellular homeostasis [31]. Previous studies have revealed that iron metabolism that induces ferritinophagy also promotes ferroptosis [25,32]; however, both mitophagy and ferritinophagy induce changes in iron content, ROS, and lipid peroxidation in the process of ferroptosis. Mitophagy is differentiated from ferritinophagy as follows [33]: 1) mitophagy is caused by hypoxia, mitochondrial damage, or nonfermentable carbon source, while ferritinophagy is induced by iron insufficiency; 2) the former is characterized by damaged mitochondria (Ub) or superfluous mitochondria, and the later by the decrease of Ferritin; 3) the receptors of mitophagy are PINK1, SQSTM1, NBR1, PHB2, etc., while the receptor of ferritinophagy is NCOA4. In this study, iron overload induced the accumulation of ROS mediated the mitochondria damage and mitophagy. This induced the increase of lipid peroxidation. Lipid metabolism modulates the formation of autophagic membrane structures via promoting the degradation of various substances within the cell, such as the release of cyto C, which, in turn, catalyzes membrane lipid peroxidation [34,35]. The results of cyto C further induce cell death. Although mitophagy may induce other forms of cell death such as apoptosis, pyroptosis, and necrosis, studies on mitophagy in ferroptosis are still limited.

Ferroptosis is a form of cell death that is induced by the accumulation of lipid peroxidation products and lethal ROS derived from iron metabolism [6–8]. Iron overload induces ferroptosis via increased release of ROS through Fenton reaction, which is characterized by the release of Fe^2+^ from the endosome into a labile iron pool in the cytoplasm [7,8]. The increase in iron uptake and decrease in iron storage contributes to excessive iron content and subsequent ferroptosis [6–10]. The mitochondrial respiratory chain and NADPH oxidases of the NOX family are major sources of ROS in human cardiomyocytes, neuronal cells, and keratinocytes [36–38]. As a key member of the NOX family, NOX1 increases the release of ROS and facilitates ferroptosis, which further exacerbates plasmodium liver stage infection [39]. GKT137831, an inhibitor of NOX1, reduces Erastin-induced ROS ferroptosis of rhabdomyosarcoma cells [40]. In this study, TFRC recruited NOX1 to PINK1 to induce the accumulation of ROS and mitophagy. Moreover, ROS reacts with the polyunsaturated fatty acids (PUFAs) of lipid membranes, to confer lipid peroxidation. ACSL4, as a major component of lipid metabolism, acylates arachidonic acid after GPX4 knockdown, which is necessary for ferroptosis. Yang et al. also demonstrated that continuous accumulation of ROS decreases the level of GSH and expression of GPX4. Numerous studies reveal that GPX4 is observed to be downregulated in response to ROS or ferroptosis inducers (such as RSL3, erastin, etc.) [18,41,42]. In this study, iron metabolism activated ROS signaling and decreased the expression of GPX4. These results suggest that lipophilic radical traps are the crucial regulators in the genetic depletion of GPX4 and the accumulation of lipid ROS [18,43]. This decrease of GPX4 in KGN cells can be the Achilles’ heel for PCOS.

## Conclusion

FAC-activated TFRC increases the uptake of iron, which promotes the accumulation of ROS and depletion of GSH. TFRC-activated signaling of NOX1 and PINK1 induces mitophagy and GSH-depletion-induced GPX4 inactivation, which is required for the ferroptosis of KGN cells. Therefore, targeting TFRC/PINK1/ACSL4/GPX4 through TFRC activation may be a potential target for PCOS.

## Highlights

(1) Iron metabolism modulates the cellular functions of KGN cells

(2) FAC activates TFRC to induce iron metabolism

(3) The activation of TFRC mediates ROS-induced mitochondrial damage and mitophagy

(4) Iron overload induced mitochondrial damage promotes lipid peroxidation and ferroptosis of KGN cells.
